# Later Midline Shift Is Associated with Better Outcomes after Large Middle Cerebral Artery Stroke

**DOI:** 10.21203/rs.3.rs-4189278/v1

**Published:** 2024-04-18

**Authors:** Jonathan J Song, Rebecca A. Stafford, Jack E. Pohlmann, Ivy So Yeon Kim, Maanyatha Cheekati, Sydney Dennison, Benjamin Brush, Stefanos Chatzidakis, Qiuxi Huang, Stelios M. Smirnakis, Emily J. Gilmore, Shariq Mohammed, Mohamad Abdalkader, Emelia J. Benjamin, Josée Dupuis, David M. Greer, Charlene J. Ong

**Affiliations:** Boston University Chobanian & Avedisian School of Medicine; Department of Neurology, Boston Medical Center; Department of Neurology, Boston Medical Center; Department of Neurology, Boston Medical Center; Department of Epidemiology, Boston University School of Public Health; Department of Epidemiology, Boston University School of Public Health; Department of Neurology, NYU Langone Medical Center; Department of Neurology, Brigham & Women’s Hospital; Department of Neurology, Jamaica Plain Veterans Administration Medical Center; Department of Neurology, Brigham & Women’s Hospital; Department of Neurology, Yale School of Medicine; Department of Biostatistics, Boston University School of Public Health; Department of Radiology, Boston Medical Center; Department of Epidemiology, Boston University School of Public Health; Department of Biostatistics, Boston University School of Public Health; Boston University Chobanian & Avedisian School of Medicine; Boston University Chobanian & Avedisian School of Medicine

**Keywords:** Midline shift, Middle cerebral artery, Cerebral edema, Timing, Mass effect, Swelling

## Abstract

**Background/Objective:**

Space occupying cerebral edema is the most feared early complication after large ischemic stroke, occurring in up to 30% of patients with middle cerebral artery (MCA) occlusion, and is reported to peak 2-4 days after injury. Little is known about the factors and outcomes associated with peak edema timing, especially when it occurs after 96 hours. We aimed to characterize differences between patients who experienced maximum midline shift (MLS) or decompressive hemicraniectomy (DHC) in the acute (<48 hours), average (48-96 hours), and subacute (>96 hours) groups and determine whether patients with subacute peak edema timing have improved discharge dispositions.

**Methods:**

We performed a two-center, retrospective study of patients with ≥1/2 MCA territory infarct and MLS. We constructed a multivariable model to test the association of subacute peak edema and favorable discharge disposition, adjusting for age, admission Alberta Stroke Program Early CT Score (ASPECTS), National Institute of Health Stroke Scale (NIHSS), acute thrombolytic intervention, cerebral atrophy, maximum MLS, parenchymal hemorrhagic transformation, DHC, and osmotic therapy receipt.

**Results:**

Of 321 eligible patients with MLS, 32%, 36%, and 32% experienced acute, average, and subacute peak edema. Subacute peak edema was significantly associated with higher odds of favorable discharge than non-subacute swelling, adjusting for confounders (aOR, 1.85; 95% CI, 1.05-3.31).

**Conclusions:**

Subacute peak edema after large MCA stroke is associated with better discharge disposition compared to earlier peak edema courses. Understanding how the timing of cerebral edema affects risk of unfavorable discharge has important implications for treatment decisions and prognostication.

## INTRODUCTION

Space occupying edema after large Middle Cerebral Artery (**MCA**) stroke is a feared complication and can occur in up to 30% of patients.^1^ Injured membrane transporters in ischemic neurons cause cells to swell with sodium and water, leading to midline shift (**MLS**) of supratentorial brain parenchyma, and in severe cases, compression or frank herniation. Resultant dysfunction and secondary injury cause neurologic deterioration, further damage, and death in 20-80% of patients, depending on surgical intervention.^2,3^

Historically, literature reports that cerebral edema occurs within the first week post-stroke, peaking between 2-4 days.^2,4^ However, cases of fulminant edema leading to deterioration have been reported prior to 48 hours and beyond 14 days.^4,5^ Early, severe peak cerebral edema is described as “malignant,” has a high mortality,^2,6^ and is an indication for life-saving surgical interventions (decompressive hemicraniectomy [**DHC**]). It is unclear to what extent timing of maximum swelling is associated with clinical outcomes. Prospective trials from ischemic stroke patients undergoing DHC have not typically included patients who experience neurologic deterioration after 96 hours.^7^ Most landmark studies on the natural progression of cerebral edema were performed on small cohorts prior to the use of modern acute stroke interventions (Supplementary Methods). To date, there has been little focus on the characteristics and course of patients who have late peak cerebral edema (more than 96 hours), limiting the study of a potential association between the edema trajectory (i.e., how fast swelling occurs) and important clinical outcomes.

## AIMS/HYPOTHESES

Understanding the association of edema timing and trajectory with outcome is clinically relevant as it may inform both prognostication and treatment decisions. This study aims to: 1) characterize differences between patients who experienced peak MLS or DHC in the acute (<48 hours), average (48-96 hours), and subacute (>96 hours) groups and; 2) determine whether patients with subacute peak edema have better discharge disposition compared to those with acute or average peak edema. We further sought to explore the association that edema trajectory had on disposition. We hypothesized that patients with subacute peak edema are more likely to have favorable discharge disposition compared to patients with acute or average peak edema.

## METHODS

### Study Population

**Figure 1:**
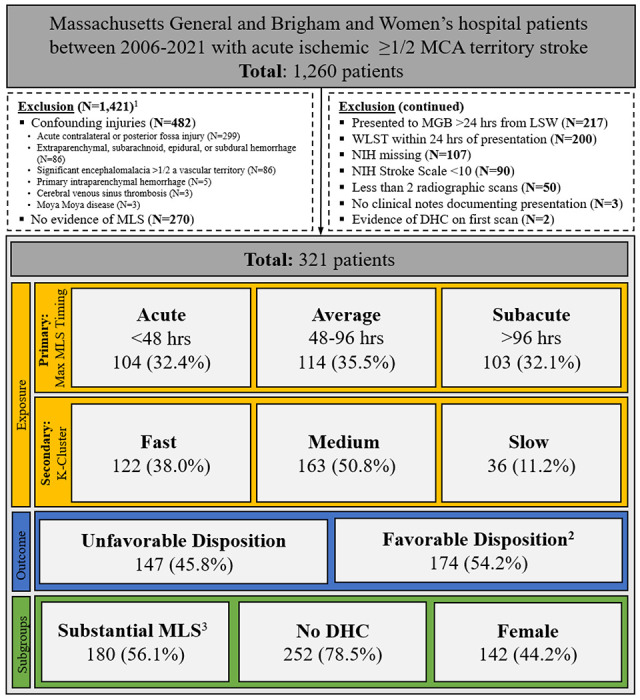
Flow Diagram of Selection of Study Sample MCA: Middle Cerebral Artery; MLS: Midline Shift; LSW: Last Seen Well; WLST: Withdrawal of Life-Sustaining Therapy; NIH: National Institute of Health; DHC: Decompressive Hemicraniectomy ^1^Patients may meet more than one exclusion criteria ^2^Favorable disposition defined as discharge to home/rehab ^3^Midline Shift ≥5mm at septum pellucidum

We conducted a two-center, retrospective cohort study of adult patients with large MCA stroke who presented within 24 hours of last seen well admitted to Brigham and Women’s Hospital and Massachusetts General Hospital between 2006 and 2021. Included patients had a National Institute of Health Stroke Scale score (**NIHSS**) >10 (post medical thrombolysis or mechanical thrombectomyif applicable), and measurable radiographic MLS during their admission. We screened for acute MCA stroke using a previously described, highly sensitive Natural Language Processing algorithm.^8^ All identified patients were confirmed to have stroke size 1/2 of the MCA territory using direct visualization of radiology images obtained within 24 hours by trained M.D. members of the research team (SC, CJO). Key exclusion criteria included patients with fewer than two radiographic images, missing NIHSS documentation, DHC prior to imaging, or life-sustaining therapy withdrawal within the first 24 hours (Figure 1).

### Data Collection

We collected clinical information from the electronic medical record using the Research Data Patient Registry and Enterprise Data Warehouse. Imaging related variables including hemorrhagic transformation, admission Alberta Stroke Program Early CT Score (**ASPECTS**), cerebral atrophy, and MLS were determined by a trained team member (SC, BB, CJO) after establishing at least 70% agreement for ordinal/categorical variables or <0.5mm mean error for continuous variables. Instances in which there was a discrepancy regarding radiographic features underwent a third review (CJO, BB) and were discussed to reach consensus. Missing values information can be found in **Supplementary Table 1**. Detailed information on data collection is included in the Supplementary Methods.We prepared the report according to Strengthening the Reporting of Observational Studies in Epidemiology reporting guidelines.^9^

### Outcomes

The primary outcome was favorable disposition, defined as discharge to home or acute rehab. Unfavorable disposition was defined as discharge to long-term care, hospice, or all-cause death. Because of the prevalence of poor functional outcomes at discharge,^10^we also used the exploratory outcome of all-cause death or hospice.

### Statistical Analysis

#### Peak Cerebral Edema Timing Analysis

We defined subacute peak edema as time to peak MLS or DHC >96 hours because this timeframe includes a significant proportion of our study participants (32.1%). Moreover, the 96-hour mark aligns with the latest time frame applied in clinical trials for DHC.^7^ Patients were further sorted into acute (<48 hours) and average (48-96 hours) peak edema categories, in line with the 48-hour benchmark cited in medical literature as the threshold for clear benefits of surgical intervention for DHC compared to medical treatment alone.^7^

We summarized patient characteristics with means and standard deviations or frequencies and proportions as appropriate. We used chi-square tests or one-way analyses of variance to test for differences between the peak edema categories. We performed preliminary analyses of baseline risk factors for hypothesis generation, using subacute peak edema as our main exposure and adjusting for age and sex.

For our primary hypothesis, we used multivariable logistic regression to test the association of favorable patient disposition and subacute cerebral edema (dichotomously) adjusting for covariates. The covariates were selected for their association with mass effect and mortality by reviewing the literature. They included age, admission ASPECTS, NIHSS, acute thrombolytic intervention, moderate or severe atrophy, maximum MLS, parenchymal hemorrhagic transformation, DHC, and receipt of osmotic therapy (**Supplementary Table 2**).

To further investigate the association of MLS timing and outcomes for patients belonging to certain subgroups, we performed various sensitivity analyses. Our first subgroup was substantial MLS ≥ 5mm, which has been used in the literature previously as a marker of severe edema.^4,11^ To address confounding effects associated with heterogeneous clinical decision-making regarding the timing of DHC, we performed a second subgroup analysis excluding patients with DHC. Finally, because we hypothesized that differences in inflammatory responses and anatomic differences between men and women may modify the extent to which patients remain edematous, we stratified our analyses by sex. We also explored treating peak edema timing as a three-factor variable, comparing the subacute peak edema group, as the reference group, to acute and average groups individually and adjusting for covariates.

#### Exploratory Trajectory Analysis

To further examine the role radiographic edema has on outcome trajectory (as opposed to absolute timing of peak edema), we conducted K-means clustering to categorize patients into three different peak edema trajectories of MLS. We used a K-means clustering algorithm to separate patients into three different groups (fast, medium, and slow) based on the “trajectory” slope calculated by joining a line from last seen well (time 0) to time of maximum MLS or DHC (**Supplementary Figure 1**).

We used exploratory multivariable modeling to compare whether fast trajectory (treated dichotomously and as a three-factor variable with slow trajectory as the reference) was inversely associated with favorable disposition, adjusting for covariates. We further explored whether slow trajectories were associated with favorable discharge disposition.

We report adjusted Odds Ratios (**aOR**) with 95% confidence intervals (**CI**). We set the two-tailed significance threshold at a=0.05 for all hypotheses. The study was approved to be conducted by the Massachusetts General Brigham Institutional Review Board (2017P002564) and informed consent was not required. Further statistical details and details regarding missing data are available in Supplementary Methods.

## RESULTS

Table 1 describes admission characteristics of the 321 eligible patients after exclusion criteria were applied. Mean age was 63.8 ±14.9 years and 142 were female (44.2%). Stroke was right-sided in 170 (53.0%) patients. Mean NIHSS was 18.4 ±4.6 and mean admission ASPECTS was 4.4 ±2.9. Of all patients, 146 (45.5%) received intravenous thrombolysis and 79 (24.6%) underwent mechanical thrombectomy. Average maximum MLS was 6.7 mm ±4.2. One-hundred and seventy-four patients (54.2%) had a favorable discharge disposition (Table 2).

**Figure 2:**
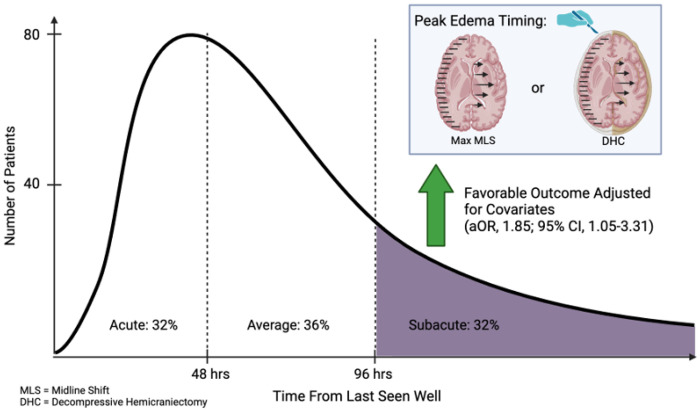
Peak Edema Timing Histogram and Study Schematic MLS: Midline Shift; DHC: Decompressive Hemicraniectomy; aOR: adjusted Odds Ratio; CI: Confidence Interval. Created with BioRender.com

One-hundred and four (32.4%), 114 (35.5%), and 103 (32.1%) of patients experienced acute, average, and subacute peak edema, respectively (Figure 2). Of the patients who experienced acute peak edema, 18 (17.3%) experienced peak MLS and 9 (8.7%) underwent DHC within 24 hours. Subacute peak edema groups had more frequent use of dual antiplatelet therapy, higher admission ASPECTS, and lower admission NIHSS (Table 1). There was a significant difference in the prevalence of hemorrhagic transformation across groups. Maximum MLS did not differ between groups (Table 2). Additional characteristics of peak edema groups and preliminary age and sex adjusted associations can be found in **Supplementary Tables 3 and 4**.

**Figure 3:**
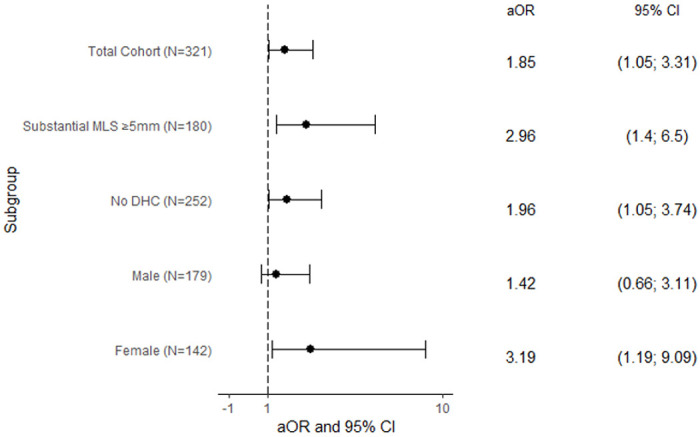
Multivariable Model – Subacute Peak Edema Timing aOR: adjusted Odds Ratio; CI: confidence interval; MLS: Midline shift; DHC: Decompressive hemicraniectomy; ASPECTS: Alberta Stroke Program Early CT Score; NIHSS: National Institute of Health Stroke Scale Multivariable logistic regression model of subacute peak edema timing adjusting for age, admission ASPECTS, NIHSS, acute thrombolytic intervention, moderate/severe atrophy, maximum MLS, parenchymal hemorrhagic transformation, DHC, and receipt of osmotic therapy in all models, with maximum MLS and DHC excluded in their respective subgroups

In the final model, subacute peak edema was associated with increased odds of favorable disposition (aOR, 1.85; 95% CI, 1.05-3.31) (Figure 3) and decreased odds of the secondary outcome, combined all-cause death and discharge to hospice (aOR, 0.35; 95% CI, 0.17-0.68) (**Supplementary Table 5**). When treated as a three-factor variable, the acute peak edema group had statistically significant decreased odds of favorable disposition compared to the subacute peak edema group (aOR, 0.50; 95% CI, 0.25-0.98) (**Supplementary Table 6**).

Subacute peak edema was experienced by 63 (35.0%) patients in the subgroup with substantial MLS ≥ 5mm (n=180), and by 96 (38.1%) patients in the subgroup without DHC (n=252). We found the direct association with favorable disposition remained significant in both subgroups of substantial MLS ≥ 5mm (aOR, 2.96; 95% CI, 1.40-6.50) and patients without DHC (aOR 1.96; 95% CI, 1.05-3.74) (Figure 3).

When stratifying by sex, there was no significant difference between the rate of subacute peak edema in women versus men (27% v. 36%, p=0.09). Subacute edema timing remained significantly associated with favorable disposition in women (aOR, 3.19; 95% CI, 1.19-9.09), but not in men (Figure 3).

In our exploratory analysis on peak edema trajectories, 36 (11.2%) patients were classified as having a slow trajectory, as opposed to 163 (50.8%) who were classified as medium and 122 (38.0%) as fast. Most patients with acute and average peak edema timing had fast (77.9%) and medium (64.9%) edema trajectories, respectively. Patients with subacute peak edema tended to have medium trajectories (66.0%), with fewer classified as slow (27.2%) (Table 2). A slow edema trajectory was not significantly associated with discharge disposition in our trajectory analysis (**Supplementary Tables 6 and 7**).

## DISCUSSION

Space occupying cerebral edema is a well-known and feared complication of MCA ischemic stroke. However, to our knowledge no studies focus on the role that subacute peak edema has on outcome. Moreover, fundamental questions regarding what factors lead some patients to swell early rather than later remain unanswered. In our cohort of 321 patients, one-third experienced peak edema experience *after* 96 hours. The high percentage of patients reaching peak swelling beyond the typical 2–4-day period underscores the need to more thoroughly describe the traits of this frequently ignored group. Our study finds that subacute peak edema timing is significantly associated with better discharge disposition after adjusting for confounders.

Our study’s findings corroborate the clinical belief that the later the onset and peak of edema following a stroke, the less life threatening it is. Nevertheless, better understanding of the factors associated with peak edema timing and its impact on patient outcomes is vital for making informed treatment choices and forecasts, particularly for the one-third of patients with subacute trajectories who currently lack sufficient guidelines for surgical or medical intervention.^1,12,13^ The 2021 European Stroke Guidelines recommend that “surgical decompression should be considered on a case-by-case basis later than 48 hours based on clinical grounds if death due to herniation appeared likely.”^13^ However, little is known about how frequently severe swelling leads to poor outcome, especially after 96 hours. Our observation that subacute peak edema presents a lower risk than acute edema likely reflects common clinical observations and justifies the exclusion of patients who present after 96 hours from clinical trials.^3^

We observed that subacute peak edema was associated with higher ASPECTS and lower NIHSS on admission. These associations suggest that patients with subacute peak edema may have had smaller stroke volumes, leading to overall better outcomes. Nonetheless, the peak MLS was comparable across acute, average, and subacute groups, indicating that the extent of the mass effect was similar among all patient categories. It is important to note that subacute peak edema is not entirely benign– 41 (39.8%) patients with a subacute course died or went to hospice or long-term care, and seven (6.8%) patients received DHC, illustrating that in certain cases, subacute clinical deterioration still necessitates extreme surgical intervention.

Our analysis revealed that patients experiencing subacute peak edema more often received dual antiplatelet therapy, developed petechial hemorrhage, and were less prone to parenchymal hemorrhagic transformation. Possible reasons for our observed associations may include later edema development in patients with intracranial atherosclerosis or ischemic cardiomyopathies requiring dual antiplatelet use. Patients with petechial hemorrhage may experience later, mild edema due to potential mild inflammatory effects of the petechial hemorrhage, unlike the acute peak edema timing we found to be associated with parenchymal hemorrhage.

Differences in the relation between subacute edema timing and discharge disposition between men and women were intriguing and unexpected. However, subacute peak edema timing was associated with significantly increased odds of favorable discharge disposition only in women, even though men were more likely to achieve a favorable disposition than women (61% v. 45%, p<0.01) (**Supplementary Tables 8-10**). The underlying causes for this observation remain uncertain, but they might be related to sex-based immunological differences affecting the incidence of severe versus mild edema development.^14^ Because we found differing results in women versus men, we conducted a secondary analysis formally testing the interaction between sex and subacute edema timing. We found no statistically significant evidence of an interaction, although the confidence interval is large, and the lack of significant interaction may be due to low power (**Supplementary Table 11**).

The absence of a relationship between edema trajectory and disposition was counter to our expectation that fast edema trajectories are associated with worse outcomes, and conversely, that slower trajectories are less malignant. Our initial expectations posited that trajectory analysis would enrich our understanding of the cumulative severity and progression MLS by capturing a broader spectrum of clinical scenarios. However, this approach may inadvertently conflate distinct patient groups–such as those experiencing a minor peak MLS shortly after onset and those developing a significant peak MLS over an extended period–which likely represent different disease trajectories with disparate outcomes. Moreover, trajectories are even more likely to be subject to biases conferred by heterogenous imaging decisions than the simpler measurement of timing of peak MLS. Our finding that the majority (77.9%) of fast-progressing edema cases developed within 48 hours supports the recognition of a distinct, very early-onset ‘malignant’ edema phenotype, with rapid swelling occurring sooner than the traditionally expected 48–72-hour timeframe. Additionally, the detection of 6.8% of fast-progressing edema cases in the subacute peak group underscores that while uncommon, significant MLS is still a risk factor approaching a week of longer post-stroke.

Limitations of our study include its retrospective nature and potential biases conferred by the heterogeneity in decision making across clinicians. Variability in clinicians’ choices about when and whether to obtain imaging could have introduced a bias in the detection and timing of MLS observations. Additionally, cases where treatment was discontinued, or where DHC was performed without a preceding CT scan may lead to inaccuracies in determining the timing of peak edema. MLS is a commonly used quantitative marker of worsening cerebral edema but does not account for mass effect vectors that may compress the brainstem or cause herniation more caudally. While we adjusted for hypothesized confounders, residual confounding cannot be excluded. Our cohort came from two hospital systems from a single region in which much of the population is largely White, and generalizability is uncertain. While our cohort of patients with longitudinal data on MLS is among the largest of its kind, our sample size is still relatively small and may not have been large enough to detect significant associations between our outcomes and exposures.

Despite these limitations, we offer comprehensive and quantitative data on the incidence of peak edema in patients with large ischemic MCA stroke while adjusting for important confounders and demonstrate that patients with subacute swelling are more likely to have favorable disposition at discharge than groups with earlier peak edema windows. Phenotyping these groups is important to identify potential clinically relevant markers to predict edema course and determine appropriate treatment pathways.

## CONCLUSION

Patients with subacute peak edema 96 hours or later after a large MCA stroke have better discharge dispositions than those with acute or average peak edema timing, particularly among women. We did not find that edema trajectory was associated with disposition, but our study may not have been appropriately powered to detect an effect. Further investigation of patients with different peak edema timing may help inform treatment decisions, clinical trials, and different underlying mechanisms related to edema formation in this critically ill population, especially in those that experience later edema onset.

## Figures and Tables

**Table 1.** Baseline Characteristics

Variable	Total N=321	Peak Edema Timing			
		Acute	Average	Subacute	p
		N=104 (32.4)	N=114 (35.5)	N=103 (32.1)	
**Demographics**					
Age	63.8 ±14.9	61.7 ±15.5	63.8 ±14.8	66.0 ±14.1	0.11
Sex (Female)	142 (44.2)	51 (49.0)	53 (46.5)	38 (36.9)	0.18
Race	-	-	-	-	0.66
White	237 (73.8)	81 (77.9)	83 (72.8)	73 (70.9)	-
Black	29 (9.0)	6 (5.8)	12 (10.5)	11 (10.7)	-
Asian	16 (5.0)	7 (6.7)	4 (3.5)	5 (4.9)	-
Other/NA	39 (12.1)	10 (9.6)	15 (13.2)	14 (13.6)	-
**Prior Medical History**					
Hypertension	216 (67.3)	74 (71.2)	70 (61.4)	72 (69.9)	0.24
Atrial Fibrillation	130 (40.5)	40 (38.5)	47 (41.2)	43 (41.7)	0.87
Congestive Heart Failure	82 (25.5)	19 (18.3)	29 (25.4)	34 (33.0)	0.05
Prior Stroke	26 (8.1)	9 (8.7)	6 (5.3)	11 (10.7)	0.33
**Home Medications**					
Antiplatelets	78 (24.3)	21 (20.2)	28 (24.6)	29 (28.2)	0.41
Dual Antiplatelets	8 (2.5)	2 (1.9)	0 (0)	6 (5.8)	0.02
**Clinical Stroke Characteristics and Acute Interventions**					
Inpatient Stroke	16 (5.0)	4 (3.8)	4 (3.5)	8 (7.8)	0.29
Stroke Side (Right)	170 (53.0)	56 (53.8)	52 (45.6)	62 (60.2)	0.10
Admission NIHSS	18.4 ±4.6	19.3 ±5.0	18.5 ±4.2	17.5 ±4.3	0.02
Large Vessel Occlusion	166 (51.7)	51 (49.0)	57 (50.0)	58 (56.3)	0.52
Large Vessel Occlusion Location^1^	-	-	-	-	0.18
Internal Carotid Artery	80 (48.8)	19 (38.0)	27 (48.2)	34 (58.6)	-
M1	57 (34.8)	21 (42.0)	22 (39.3)	14 (24.1)	-
M2, M3, or M4	27 (16.5)	10 (20.0)	7 (12.5)	10 (17.2)	-
ACA Involvement	92 (28.7)	32 (30.8)	38 (33.3)	22 (21.4)	0.13
Intravenous Thrombolysis	146 (45.5)	44 (42.3)	56 (49.1)	46 (44.7)	0.59
Mechanical Thrombectomy^1^	79 (24.6)	25 (24.0)	28 (24.6)	26 (25.2)	0.98
≥ TICI 2b	41 (51.9)	14 (56.0)	15 (53.5)	12 (46.1)	0.92
**Radiographic Stroke Characteristics**					
Moderate/Severe Cortical Atrophy	112 (34.9)	34 (32.7)	37 (32.5)	41 (39.8)	0.45
Admission ASPECTS	4.4 ±2.9	3.9 ±2.8	4.4 ±3.0	4.9 ±2.9	0.05

NIHSS: National Institute of Health Stroke Scale; M1: Sphenoidal segment of the middle cerebral artery; M2: Insular segment of middle cerebral artery; M3: Opercular segment of middle cerebral artery; M4: Cortical segment of middle cerebral artery; ACA: Anterior cerebral artery; TICI: Thrombolysis in cerebral infarction scale; ASPECTS: Alberta Stroke Program Early CT Score

Parentheses indicate percentages for categorical variables; ± indicate standard deviations for continuous variables.

1 Subgroup frequencies calculated in context of having the header label of interest

**Table 2.** Hospitalization Events and Outcomes

Variable	Total N=321	Peak Edema Timing			
		Acute N=104 (32.4)	Average N=114 (35.5)	Subacute N=103 (32.1)	p
**Mass Effect**					
Hemorrhagic Transformation					
No Hemorrhage	87 (27.1)	37 (35.6)	31 (27.2)	19 (18.4)	0.02
Parenchymal Hemorrhage	42 (13.1)	21 (20.2)	12 (10.5)	9 (8.7)	0.03
Petechial Hemorrhage	216 (67.3)	56 (53.8)	79 (69.3)	81 (78.6)	<0.01
Midline Shift (MLS)					
Substantial MLS	180 (56.1)	54 (51.9)	63 (55.3)	63 (61.2)	0.40
Maximum MLS (mm)	6.7 ±4.2	6.4 ±4.2	6.4 ±3.9	7.3 ±4.5	0.22
Time to Maximum MLS (hr)	87.1 ±60.9	39.3 ±34.1	69.7 ±21.0	154.7 ±51.9	<0.01
Pineal Gland Shift (PGS)					
PGS ≥ 4mm	122 (38.0)	32 (30.8)	42 (36.8)	48 (46.6)	0.06
Maximum PGS (mm)	3.8 ±2.5	3.5 ±2.3	3.7 ±2.3	4.3 ±2.9	0.06
Time to Maximum PGS (hr)	82.7 ±66.3	46.6 ±56.5	69.4 ±42.4	133.7 ±66.5	<0.01
Decompressive Hemicraniectomy	69 (21.5)	40 (38.5)	22 (19.3)	7 (6.8)	<0.01
Osmotic Therapy	149 (46.4)	56 (53.8)	49 (43.0)	44 (42.7)	0.18
Peak Edema Trajectory Cluster	-	-	-	-	<0.01
Fast	122 (38.0)	81 (77.9)	34 (29.8)	7 (6.8)	-
Medium	163 (50.8)	21 (20.2)	74 (64.9)	68 (66.0)	-
Slow	36 (11.2)	2 (1.9)	6 (5.3)	28 (27.2)	-
**Discharge Outcomes**					
Unfavorable Disposition	147 (45.8)	52 (50.0)	54 (47.4)	41 (39.8)	0.31
Favorable Disposition	174 (54.2)	52 (50.0)	60 (52.6)	62 (60.2)	0.31

Parentheses indicate percentages for categorical variables; ± indicate standard deviations for continuous variables.

## Data Availability

Data supporting this study are available upon request.

## References

[1] Wijdicks EF, Sheth KN, Carter BS, Recommendations for the management of cerebral and cerebellar infarction with swelling: a statement for healthcare professionals from the American Heart Association/American Stroke Association. Stroke 2014;45(4):1222–38. DOI: 10.1161/01.str.0000441965.15164.d6. 24481970

[2] Hacke W, Schwab S, Horn M, Spranger M, De Georgia M, von Kummer R. ‘Malignant’ middle cerebral artery territory infarction: clinical course and prognostic signs. Arch Neurol 1996;53(4):309–15. DOI: 10.1001/archneur.1996.00550040037012. 8929152

[3] Hofmeijer J, Kappelle LJ, Algra A, Surgical decompression for space-occupying cerebral infarction (the Hemicraniectomy After Middle Cerebral Artery infarction with Life-threatening Edema Trial [HAMLET]): a multicentre, open, randomised trial. Lancet Neurol 2009;8(4):326–33. DOI: 10.1016/S1474-4422(09)70047-X. 19269254

[4] Pullicino PM, Alexandrov AV, Shelton JA, Alexandrova NA, Smurawska LT, Norris JW. Mass effect and death from severe acute stroke. Neurology 1997;49(4):1090–5. DOI: 10.1212/wnl.49.4.1090. 9339695

[5] Adams JH, Graham DI. Twelve cases of fatal cerebral infarction due to arterial occlusion in the absence of atheromatous stenosis or embolism. Journal of Neurology, Neurosurgery & Psychiatry 1967;30(6):479–488. DOI: 10.1136/jnnp.30.6.479. 5583576 PMC496237

[6] Berrouschot J, Sterker M, Bettin S, Koster J, Schneider D. Mortality of space-occupying (‘malignant’) middle cerebral artery infarction under conservative intensive care. Intensive Care Med 1998;24(6):620–3. DOI: 10.1007/s001340050625. 9681786

[7] Wei H, Jia FM, Yin HX, Guo ZL. Decompressive hemicraniectomy versus medical treatment of malignant middle cerebral artery infarction: a systematic review and meta-analysis. Biosci Rep 2020;40(1). DOI: 10.1042/BSR20191448. PMC6944664 31854446

[8] Ong CJ, Orfanoudaki A, Zhang R, Machine learning and natural language processing methods to identify ischemic stroke, acuity and location from radiology reports. PLOS ONE 2020;15(6):e0234908. DOI: 10.1371/journal.pone.0234908. 32559211 PMC7304623

[9] von Elm E, Altman DG, Egger M, The Strengthening the Reporting of Observational Studies in Epidemiology (STROBE) Statement: guidelines for reporting observational studies. Int J Surg 2014;12(12):1495–9. DOI: 10.1016/j.ijsu.2014.07.013. 25046131

[10] Reinink H, Juttler E, Hacke W, Surgical Decompression for Space-Occupying Hemispheric Infarction: A Systematic Review and Individual Patient Meta-analysis of Randomized Clinical Trials. JAMA Neurol 2021;78(2):208–216. DOI: 10.1001/jamaneurol.2020.3745. 33044488 PMC7551237

[11] Wu S, Yuan R, Wang Y, Early Prediction of Malignant Brain Edema After Ischemic Stroke. Stroke 2018;49(12):2918–2927. DOI: 10.1161/STROKEAHA.118.022001. 30571414

[12] Cook AM, Morgan Jones G, Hawryluk GWJ, Guidelines for the Acute Treatment of Cerebral Edema in Neurocritical Care Patients. Neurocrit Care 2020;32(3):647–666. DOI: 10.1007/s12028-020-00959-7. 32227294 PMC7272487

[13] van der Worp HB, Hofmeijer J, Juttler E, European Stroke Organisation (ESO) guidelines on the management of space-occupying brain infarction. Eur Stroke J 2021;6(2):XC–CX. DOI: 10.1177/23969873211014112. 34414308 PMC8370072

[14] Klein SL, Flanagan KL. Sex differences in immune responses. Nat Rev Immunol 2016;16(10):626–38. DOI: 10.1038/nri.2016.90. 27546235

